# RAFT Copolymerization of Vinyl Acetate and Acrylic Acid in the Selective Solvent

**DOI:** 10.3390/polym14030555

**Published:** 2022-01-29

**Authors:** Elena Yu. Kozhunova, Anna V. Plutalova, Elena V. Chernikova

**Affiliations:** 1 Faculty of Physics, Lomonosov Moscow State University, Lenin Hills, 1, bld. 2, 119991 Moscow, Russia; 2 Faculty of Chemistry, Lomonosov Moscow State University, Lenin Hills, 1, bld. 3, 119991 Moscow, Russia

**Keywords:** reversible addition-fragmentation chain transfer (RAFT) polymerization, copolymerization, gradient copolymers, self-assembly

## Abstract

Reversible addition-fragmentation chain transfer polymerization was successfully applied to the synthesis of the gradient copolymer of acrylic acid and vinyl acetate in the selective solvent. The gradient degree of the copolymer was varied by the monomer feed. The monomer conversion was found to affect the ability of the copolymer to self-assemble in aqueous solutions in narrowly dispersed micelles with an average hydrodynamic radius of about 250 nm. Furthermore, the synthesized copolymers also tended to self-assemble throughout copolymerization in the selective solvent.

## 1. Introduction

The direct synthesis of block copolymers self-assembled in the core-shell nanoparticles of various morphologies has gained significant interest over the past decade [1,2,3,4,5,6,7,8,9]. This process, known as polymerization-induced self-assembly (PISA), is based on the ability of block copolymers to self-assemble in a selective solvent. In this case, the first block is solvophilic, while the second block is solvophobic. PISA requires the use of a living or reversible-deactivation radical mechanism and may be performed using a two-stage process [7]. In the first stage, the solvophilic living polymer is prepared through solution polymerization. In the second stage, this polymer is dissolved in a thermodynamically good solvent. Then, it is chain-extended through dispersion or emulsion polymerization of the second monomer that forms a solvophobic block. Alternatively, a one-pot approach may be used. Solvophilic living polymer is prepared directly in the desired media, and the second monomer, forming a solvophobic block, is introduced in the reaction media after full consumption of the first monomer. After the solvophobic block reaches the critical length, the self-assembly of the block copolymer into nanoparticles occurs, and further growth of the solvophobic block continues inside the particles. Thus, the living polymer provides the formation of the block copolymer and stabilizes the formed nanoparticles. PISA may be performed via a stable free-radical (SFRP) mechanism, atom transfer (ATRP) mechanism, or reversible addition-fragmentation chain transfer (RAFT) mechanism [10,11,12,13,14,15].

A similar result may be achieved in the case when two monomers possess significantly different reactivity in reversible-deactivation radical copolymerization, and homopolymers formed by these monomers are solvophobic and solvophilic concerning the chosen solvent (reaction medium), respectively [16,17,18,19,20]. Depending on the absolute values of the monomer reactivity ratio, a random, gradient, block gradient, or block copolymer may form. Both factors, namely, monomer reactivity and solvent selectivity, are crucial to provoke the self-assembly of the forming copolymers. The rate of the insertion of the given monomer in the macromolecule throughout copolymerization may be controlled by several parameters: monomer feed, monomer reactivity, and the rate of gradual addition of one or both monomers in the reaction [21,22]. In the case of polar and low polarity monomers, monomer reactivity may be varied by the polarity of the solvent and polarity of the macroinitiator (or macro-RAFT agent) [23,24].

Recently ring-opening metathesis polymerization (ROMP) was employed to demonstrate that gradient copolymers could induce in situ self-assembly of conjugated polymers in organic solvents [3]. Another example is the aqueous PET-RAFT copolymerization of specific monomers such as oligo(ethylene glycol), methyl ether methacrylate (OEGMA), and diacetone acrylamide (DAAm) or OEGMA with 2-hydroxypropyl methacrylate [17]. In situ self-assembly was also observed in the TEMPO-mediated bulk copolymerization of styrene and N-vinylpyrrolidone [25]. These are a few examples of the known systems able to cause gradient polymerization-induced self-assembly.

The present research reports the gradient polymerization-induced self-assembly in the RAFT copolymerization of vinyl acetate (VAc) and acrylic acid (AA). Vinyl acetate is a “less activated” monomer, while acrylic acid is a “more activated” monomer. Thus, their activity in copolymerization is disparate: *r*_AA_ = 10, *r*_VAc_ = 0.01 in bulk, *r*_AA_ = 3.2, *r*_VAc_ = 0.05 in ethanol (abs.), *r*_AA_ = 2.6, *r*_VAc_ = 0.04 in ethanol/water (93/7 *w*/*w*) solution, *r*_AA_ = 0.96, *r*_VAc_ = 0.06 in ethanol/water (1/1 *w*/*w*) solution, and *r*_AA_ = 1.18, *r*_VAc_ = 0.04 in methanol [26,27]. We expect that choosing an appropriate selective solvent, which is thermodynamically good for polyAA and thermodynamically poor for PVAc, may provide in situ self-assembly of the formed gradient copolymer of AA and VAc which would stimulate the growth of a gradient copolymer. In addition, the formed polymer should form micelles upon dilution in a selective solvent.

## 2. Materials and Methods

### 2.1. Materials and Polymer Synthesis

Monomers—acrylic acid, vinyl acetate, and the solvents-1,4-dioxane and N, N-dimethyl formamide (DMF) of the “Acros Organics” (Fair Lawn, NJ, USA), were distilled before use. Initiator potassium persulfate (PSK, ACS reagent) and RAFT agents dibenzyl trithiocarbonate (C_6_H_5_CH_2_–S–C(=S)–S–CH_2_C_6_H_5_) and poly(ethylene glycol) methyl ether (4-cyano-4-pentanoate dodecyl trithiocarbonate) (CH_3_(OCH_2_CH_2_)_k_OC(=O)CH_2_CH_2_C(CH_3_)(CN)–S–C(=S)–S–C_12_H_25_, PEG-TC, M_n_ = 5400) purchased from “Aldrich” (St. Louis, MO, USA) were used without further purification.

RAFT copolymerization of AA and VAc was conducted according to the following general procedure. First, the RAFT agent was dissolved in 1,4-dioxane, and VAc and AA were added. After that, the solution of PSK in bidistilled water was added drop-wise on stirring to the prepared solution. The final mixture was poured into ampoules. The reaction mixtures were degassed through four freeze−pump−thaw cycles and sealed. Next, the ampoules were immersed into the thermostat pre-heated at 80 °C for the required time. Then, the samples were cooled in liquid nitrogen; polymers were dissolved in the 10-fold excess of 1,4-dioxane and dried by lyophilization in a vacuum.

The typical receipt includes 0.022 g of PEG-TC (10^−3^ mol/L) as a RAFT agent, 2.3 mL of bidistilled water, 1.6 mL of 1,4-dioxane, 1 mL of AA, 1 mL of VAc, and 0.0011 g of PSK (10^−3^ mol/L) as an initiator. Table 1 summarize the formulations of reaction mixtures for copolymerization. The total monomer conversion was determined by gravimetry, taking into account the weight of PEG-TC in the probe.

### 2.2. Instrumentation

The average molecular weights and dispersity (*Ð* = *M_w_*/*M_n_*) were determined by size exclusion chromatography (SEC). The SEC measurements were performed in DMF containing 0.1 wt% of LiBr at 50 °C with a flow rate of 1.0 mL/min using a chromatograph GPC-120 “PolymerLabs” (Hichrom Limited, Theale, UK) equipped with refractive index and with two columns PLgel 5 µm MIXED B for MW range 5 × 10^2^–1 × 10^7^. The SEC system was calibrated using narrow dispersed linear poly(methyl methacrylate) standards with MW ranging from 800 to 2 × 10^6^ g mol^−^^1^. A second-order polynomial was used to fit the log_10_*M* versus retention time dependence. All copolymers were subjected to methylation by diazomethane before analysis.

The average hydrodynamic radius *R_h_* of the particles of polymeric dispersions was determined by dynamic light scattering (DLS). DLS measurements were performed by a static/dynamic compact goniometer (DLS/SLS-5000, ALV, Langen, Germany). A HeNe laser with a power of 22 mW emitting a polarized light at λ = 633 nm was used as the incident beam. The studies were carried out at 23 °C and a scattering angle of 90°. Typical weight concentrations of the samples were 0.01 and 0.05 g/L; bidistilled water and 1,4-dioxane were used as solvents. Distributions over decay times were obtained using a nonlinear regularized inverse Laplace transformation method (CONTIN) [28].

The copolymer composition was analyzed by ^1^H NMR using the “VARIAN XR-400” spectrometer (Paolo Alto, CA, USA) at 400 MHz. The samples were prepared by dissolving the copolymers in DMSO-d_6,_ which was used as an internal standard.

## 3. Results and Discussion

### 3.1. Copolymerization of Vinyl Acetate and Acrylic Acid

**Figure 1:**
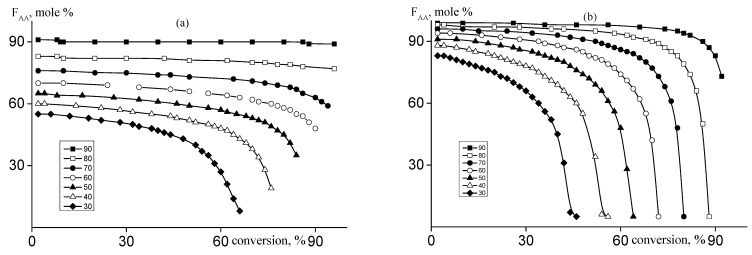
The instantaneous molar part of acrylic acid F_AA_ in copolymer as a function of the overall monomer conversion calculated for *r*_AA_ = 0.96, *r*_VAc_ = 0.06 (**a**) and *r*_AA_ = 10, *r*_VAc_ = 0.01 (**b**) at different monomer feeds f_AA_ = 30 (1), 40 (2), 50 (3), 60 (4), 70 (5), 80 (6), and 90 mole% (7).

Acrylic acid is a “more activated” monomer, unlike “less activated” vinyl acetate, independent of the solvent polarity [25]. However, its monomer reactivity ratios vary widely for copolymerization in bulk or solution. Thus, to estimate the monomer feed providing the formation of a gradient copolymer, we calculated the instantaneous molar part of acrylic acid in the copolymer as a function of the overall monomer conversion for extreme values of the known monomer reactivity ratios for this monomer pair (*r*_AA_ = 0.96, *r*_VAc_ = 0.06 and *r*_AA_ = 10, *r*_VAc_ = 0.01 [26]). The dependences of instantaneous copolymer composition on the monomer conversion at different monomer feeds are presented in Figure 1. When AA is 16 times more active in the copolymerization than VAc (*r*_AA_ = 0.96, *r*_VAc_ = 0.06, Figure 1a), the noticeable change of F_AA_ at high monomer conversions is observed at f_AA_ < 50 mole%. If the difference in the monomer reactivity is 10^3^ (Figure 1b), the noticeable change of F_AA_ is observed even at f_AA_ < 90 mole%. The decrease of AA content in the monomer feed enhances this change in both cases. In conditions of conventional radical copolymerization, the noticeable change of instantaneous copolymer composition results in the formation of copolymers with high composition heterogeneity, while in conditions of RAFT copolymerization, the formation of a gradient copolymer is expected. Thus, based on the assumption that the monomer reactivity ratio of AA and Vac, lying within the limits of the mentioned values of *r*_AA_ and *r*_VAc_, we chose monomer feeds at f_AA_ < 60 mole% for further experiments.

The performance of gradient polymerization-induced self-assembly is based on the use of the selective solvent for one of the monomers. The bulk RAFT copolymerization of AA and VAc (f_AA_ = 10 and 50 mole%) was conducted first in the presence of dibenzyl trithiocarbonate, which is more suitable for AA and less suitable for VAc [29,30]. Polyacrylic acid is insoluble in its monomer, ketones, and esters [31], including VAc, while polyvinyl acetate has good solubility in VAc and AA, as we have checked experimentally. In this case, acrylic acid being more active in the copolymerization than vinyl acetate, is consumed first and should form a low soluble product. Bulk copolymerization proceeds very rapidly and results in the formation of the product non-soluble in any organic solvent or water, indicating that the RAFT mechanism is violated. The non-soluble (cross-linked) polymer formation is probably caused by chain transfer to polymers and monomers [32,33].

Thus, the reaction media should be a good solvent for polyacrylic acid and a poor solvent or non-solvent for polyvinyl acetate. Simultaneously, the decrease of monomer concentration by diluting the mixture with the solvent should decrease the probability of the side reactions, such as transfer to polymers or intermediate radical termination that are sensitive to the concentration of propagating species. Water is the better selective solvent for the chosen monomer pair, as it is a good solvent for polyacrylic acid and is non-solvent for polyvinyl acetate. However, vinyl acetate also has low solubility in water. Therefore, a mixed solvent was used, containing water and 1,4-dioxane taken in 1.4 to 1 volume ratio. This composition corresponds to the requirement of the selective solvent. PEG-TC was used as a water-soluble RAFT agent to achieve the RAFT mechanism, and as an initiator, PSK is soluble both in water and 1,4-dioxane.

**Figure 2:**
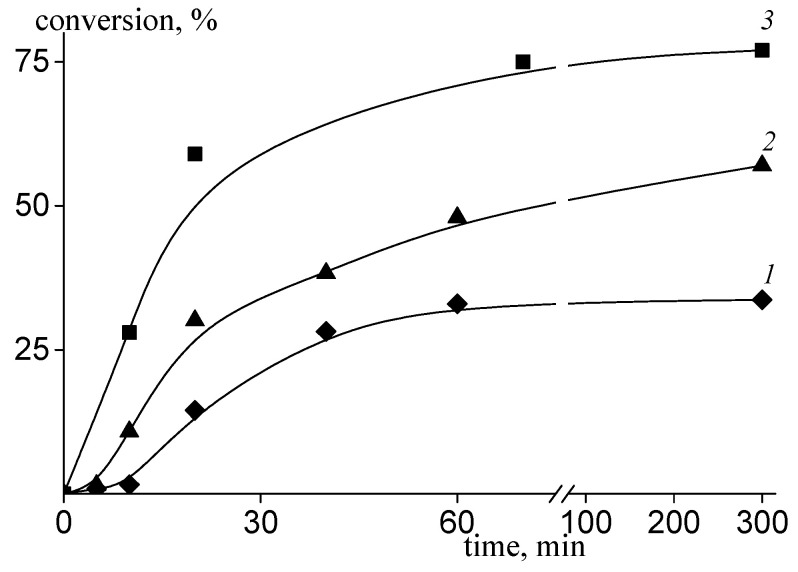
Dependence of the monomer conversion on time for RAFT copolymerization of VAc and AA conducted at 80 °C in water—1,4-dioxane mixture in the presence of PEG-TC and PSK at f_AA_ = 30 (1), 45 (2), and 57 mole% (3) in monomer feed.

Figure 2 present the time-dependent conversion for the systems containing 30, 45, and 57 mole% AA in monomer feed. For high VA content in the monomer feed, the induction period is observed on the kinetic curves. Its duration decreases, and the overall rate increases with VA content decrease in the monomer feed. This behavior is typical for the RAFT homo- and copolymerization of VA and is caused by the slow fragmentation of the intermediate radicals and their cross- or self-termination [29].

**Figure 3:**
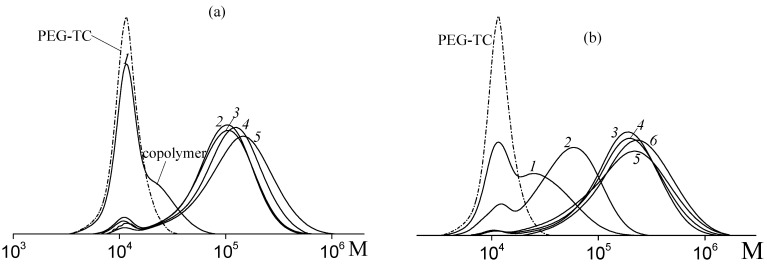

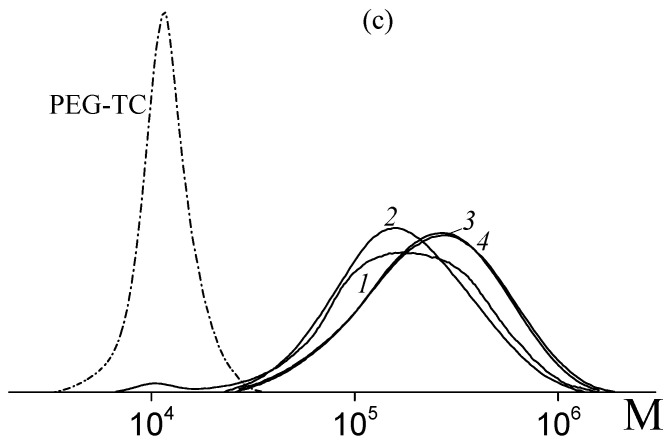
MWDs of the copolymers synthesized through RAFT copolymerization of VAc and AA at 80°C in water–1,4-dioxane mixture in the presence of PEG-TC and PSK at f_AA_ = 30 (**a**), 45 (**b**), and 57 mole% (**c**) in monomer feed. (**a**) conversion: 0.9 (1), 14.5 (2), 28.2 (3), 33.0 (4) and 33.7% (5); (**b**) conversion: 1.5 (1), 10.8 (2), 30.1 (3), 38.3 (4), 48 (5) and 57% (6); (**c**) conversion: 28 (1), 59 (2), 75 (3) and 77% (4). MWD of initial PEG-TC is given for comparison.

PEG-TC is a moderately efficient RAFT agent. Figure 3 present MWDs of the copolymers formed from various monomer feeds at different monomer conversions. At low monomer conversion, the reaction products are characterized by bimodal MWD (Figure 3a,b). The low molecular weight mode corresponds to PEG-TC, while the high molecular weight mode corresponds to the formed copolymer. Throughout copolymerization, the intensity of the mode assigned to PEG-TC rapidly decreases, and it disappears after 50–55% of monomer conversion. As a result, the mode of the grown copolymer shifts to the region of high molecular weights. This behavior is typical for the RAFT mechanism and is observed for all the studied systems. In the case of the system containing 57 mole% AA in monomer feed, copolymerization proceeds rather fast, so it becomes impossible to stop it at low or middle conversions. Thus, MWDs are mostly unimodal, as PEG-TC is fully consumed (Figure 3c).

**Figure 4:**
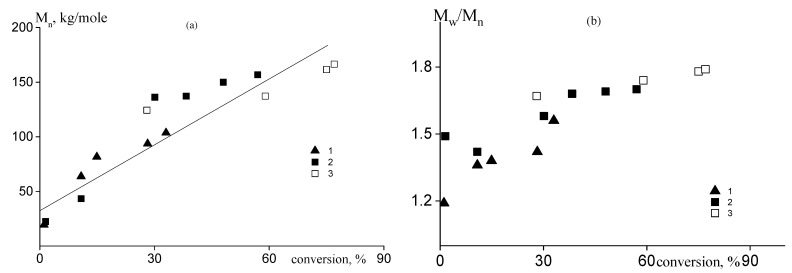
Dependence of the number-average molecular weight, M_n_ (**a**), and dispersion M_w_/M_n_ (**b**) on monomer conversion for the copolymers synthesized from the monomer feed with f_AA_ = 30 (1), 45 (2), and 57 mole% (3).

The SEC modes on the chromatograms were separated, and the molecular weight characteristics of the higher molecular weight mode corresponding to the growing copolymer were analyzed. The dependence of number-average molecular weight, M_n_ of the growing copolymers is plotted in Figure 4a; corresponding data are given in Table S1 (Supporting Information file). It is seen that M_n_ increases with the progress of monomer conversion independently of the monomer feed, confirming the RAFT mechanism of the polymerization. Dispersity of the copolymers increases with monomer conversion from 1.2 to 1.8 (Figure 4b) due to the equimolar ratio of the initiator and RAFT agent concentrations, i.e., the amount of “dead” chains increases during the course of polymerization.

**Figure 5:**
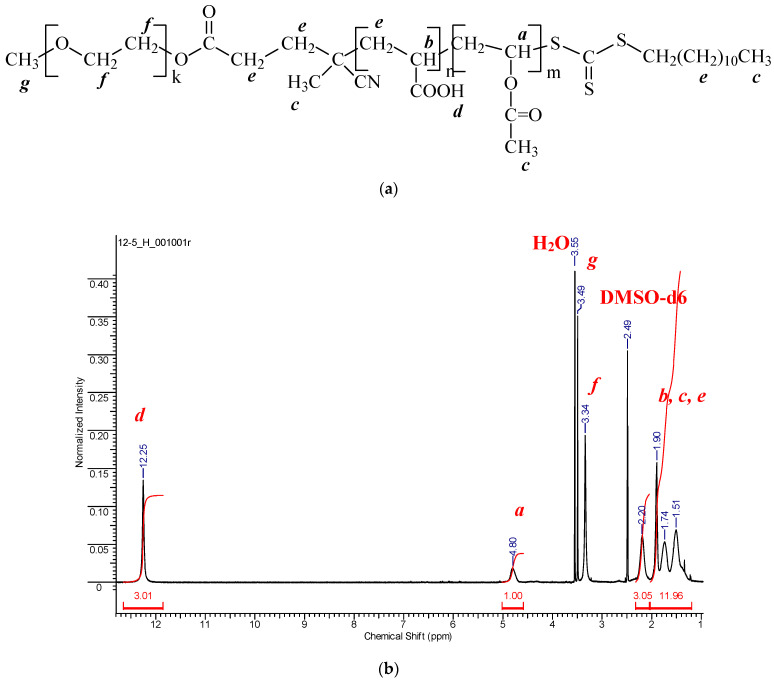
(**a**) Chemical structure of the synthesized copolymer of VAc and AA; (**b**) ^1^H NMR spectrum in DMSO-d6 of the copolymer of VAc and AA synthesized in PEG-TC mediated copolymerization from monomer feed containing 45 mole% of AA at 57% conversion.

^1^H NMR spectroscopy was used to estimate the copolymer composition. Figure 5b present the typical ^1^H NMR spectrum of the copolymer formed in DMSO-d6; other spectra are given in ESI, Figure S1. As the RAFT mechanism is achieved, the copolymer mainly has the following structure (k~100; m and n values depend on monomer feed and conversion).

The analysis of the ^1^H NMR spectra confirms the copolymer structure. The detailed assignment of the signals is given in ESI. The average copolymer composition was determined using the signals of AA units at 12.25 ppm (COOH of acrylic acid) and Vac units at 4.80 ppm (CH of the backbone). The results of the calculation of the copolymer composition are summarized in Table 2. The copolymer is enriched with AA independent of the monomer feed confirming the high activity of AA in the copolymerization compared to VAc.

Thus, we have shown that the copolymerization of AA and VAc proceeds through the RAFT mechanism and results in the formation of the diblock copolymer, which contains the short PEG block (~115 units) and the long block of the copolymer of VAc and AA (~1500–1900 units). The microstructure of the second block depends on the monomer reactivity and monomer conversion. Our preliminary estimations suppose that the gradient copolymer of VAc and AA is formed in all cases. It may also be presumed that the gradient degree for copolymer formed at f_AA_ = 30 mole% is low due to low monomer conversion. With the growth of monomer conversion, the gradient degree should increase. Thus, the copolymer formed at f_AA_ = 57 mole% is the most preferable for further investigations due to the maximum value of the achieved monomer conversion.

### 3.2. Self-Assembly

It is known that gradient copolymers in selective solvents have the pronounced ability for self-assembly similar to that of regular block copolymers with various assembled structures [34,35,36]. Sometimes, gradient copolymers are even more preferable for the micelles’ formation due to lower aggregation number, specific internal structure, and one-step polymerization process [37].

**Figure 6:**
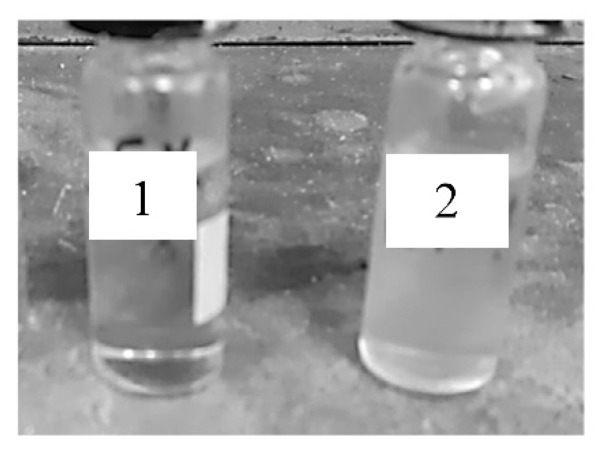
The photographs of the reaction mixtures obtained for copolymerization at f_AA_ = 57 mole% after 10 min, 28% conversion (1), and 300 min, 77% conversion (2).

In the system under study, self-organization is expected to occur directly during the polymerization process due to the self-assembly of the growing gradient copolymers under the influence of selective to the second “block” mixed 1,4-dioxane–water solvent. The PEO block’s length is short compared to the length of the grown copolymer of AA and VAc. Moreover, the PEO block is connected with hydrophilic AA units, and the product’s overall structure may be considered a gradient as it is transferred from hydrophilic to hydrophobic units. Indeed, we noted that the reaction mixtures were continuously losing their transparency and became opaque after copolymerization (Figure 6). The preliminary studies of the reaction mixture by SAXS and TEM confirmed that self-assembly occurs throughout copolymerization (ESI, Figures S2 and S3).

**Figure 7:**
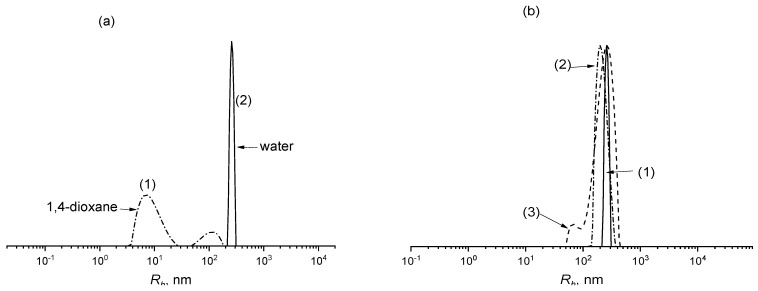
(**a**) Hydrodynamic radius *R_h_* distribution of gradient copolymer (f_AA_ = 57 mole%) in 1,4-dioxane (1) and water (2), c = 0.1 wt%; (**b**) *R_h_* distribution of gradient copolymer (f_AA_ = 57 mole%) in water at different polymer concentrations, c = 0.1 (1), 0.5 (2), and 1 wt% (3).

Moreover, self-organization is also expected for the purified gradient copolymers in selective solvents, and it is important for further applications. We investigated the ability of the synthesized products to form microstructures in 1,4-dioxane (good solvent) and water (selective solvent), using the dynamic light scattering (DLS) method. Figure 7a confirms that the behavior of the gradient copolymer of VAc and AA formed at 77% of monomer conversion (f_AA_ = 57 mole%) in solution depends strongly on the solvent nature. In 1,4-dioxane, which is a good solvent for both poly(VAc) and poly(AA) [32], the DLS method shows that macromolecules are mainly dispersed in the solution as unimers, i.e., single polymer chains. This is typical for polymers in good solvents. A small number of aggregates is also noted. The latter observation is probably explained by the immediate vicinity of the critical temperature of solubility of this copolymer in 1,4-dioxane.

However, the change of the solvent from good to selective, in our case to water, which is a good solvent for poly(AA) and poor solvent for poly(VAc) [32], reveals the formation of quite monodisperse spheres. DLS data shows a narrow peak corresponding to the sphere with a hydrodynamic radius (*R_h_*) of about 250 ± 30 nm. We suppose that it is the gradient structure responsible for the relatively high radius of the particles. Indeed, the rough estimate of the contour length of macromolecules L = Pl (P—is the polymerization degree, l—the length of the monomer unit equal to 0.25 for vinyl polymers) gives the value ca. 500 nm. The unperturbed dimensions of copolymers may be roughly estimated from the empiric equation for PAA: $\langle h^{2}\rangle {}_{\Theta }=6.7\cdot 2Pl_{CC}$ where l_CC_ is the length of a C–C bond equal to 0.154 nm [22]. Therefore, the *R_h_* of the unperturbed coil can be calculated as ${\left(R_{h}\right)}_{\Theta }=\frac{\langle h^{2}\rangle {{}_{\Theta }}^{\frac{1}{2}}}{1.5\sqrt{6}}\approx 17.5\;nm$. As is seen, the hydrodynamic radius is more than 10 times higher than the unperturbed dimensions of the individual coil and twice lower than the contour length of the macromolecule. However, the rod-like conformation of the lightly charged amphiphilic macromolecule in the selective solvent is unlikely. Thus, the obtained value of *R_h_* of the particles of gradient copolymer can be interpreted as the formation of micelles caused by hydrophobic interactions of low polar VAc units. According to the RAFT mechanism and higher activity of AA in copolymerization, the micelle consists of the hydrophilic shell formed by the short block of PEG with a gradient block of copolymer enriched with AA and the hydrophilic core formed by gradient copolymer enriched with VAc. The *R_g_/R_h_* value for the dispersion of gradient copolymer (f_AA_ = 57 mole%) in water was found to be equal to 0.47, which is usually attributed to a sphere with a more dense core and less dense corona.

Visually, the copolymer solution in 1,4-dioxane looks transparent, while in water, it is opaque without visible inhomogeneities. To confirm the stability of micelles, the DLS study in water solution was repeated at different copolymer concentrations (Figure 7b). The increase of copolymer concentration results in a slight broadening of *R_h_* distribution, while the average size of the aggregates remains similar. Thus, in the water solution, the self-assembly of the gradient copolymer occurs, resulting in the formation of stable micelles.

**Figure 8:**
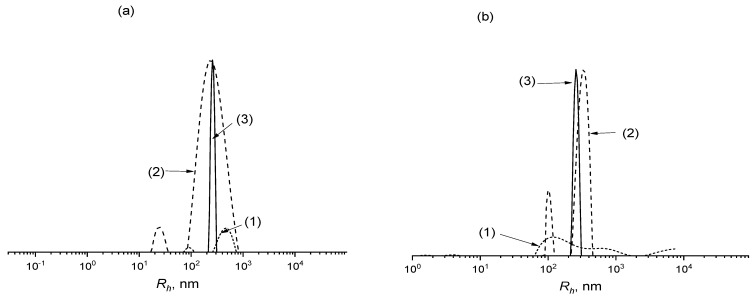
(**a**) Hydrodynamic radius *R_h_* distribution of gradient copolymer (f_AA_ = 57 mole%) formed at different conversions 28 (1), 58 (2), and 77% (3) in 0.1 wt% aqueous solution; (**b**) *R_h_* distribution of gradient copolymers formed at monomer feed f_AA_ = 30 (1), 45 (2), and 57 mole% (3) and conversion 33.7 (1), 57 (2) and 77% (3) in 0.1 wt% aqueous solution.

Due to the higher activity of AA in copolymerization with VAc, the gradient degree of the copolymer increased with the progress in the monomer conversion. Thus, the ability of the copolymer to self-assemble should was dependent on the monomer conversion. To confirm this statement, 0.1 wt% of aqueous solutions of the copolymers formed at conversions 28, 58, and 77% were analyzed by DLS (Figure 8a). The aggregates with random *R_h_* distribution were observed for the copolymer formed at 28% of the monomer conversion. This points out that the hydrophobic block was not yet sufficiently long enough to promote the micelle formation. We presume that at this stage of polymerization, the VAc monomer units were rare and randomly distributed between AA units. For the copolymer formed at 58% of monomer conversion, the weight fraction of the small aggregates fall, and the aggregates with broad *R_h_* distribution and the average *R_h_* of about 250 nm prevailed. The copolymer obtained at 77% of monomer conversion formed the micelles with a narrow *R_h_* distribution and a similar *R_h_* of about 250 nm. This copolymer had the largest number of hydrophobic units. At this conversion, the sequence of VAc units was long enough, encouraging the appearance of the micelle with a hydrophobic core and hydrophilic shell.

A similar result was observed by changing the monomer feed (Figure 8b). The decrease of the AA content in the monomer feed should increase the gradient degree. However, in this case, the monomer conversion was noticeably reduced. As a result, the ability of the copolymer to self-assemble in water was decreased with the reduction of hydrophilic AA content in the monomer feed. The copolymer synthesized from f_AA_ = 30 mole% at 33.7% conversion could not form distinct self-assembled structures. That could be explained by a mostly random distribution of AA and VAc units in the grown copolymer. The copolymer synthesized at higher conversion and AA content (57% and f_AA_ = 45 mole%) exhibited the ability to self-assemble in aqueous solution; however, it formed aggregates with bimodal *R_h_* distribution probably due to the formation of micelles and intermolecular aggregates caused by low gradient degree. Finally, the copolymer obtained at f_AA_ = 57 mole% and 77% conversion was characterized by the narrowest size distribution.

It should be noted that the obtained copolymers also showed a tendency to form microstructures in the mixed solvent “water/dioxane” as that was used in the polymerization process. The example of hydrodynamic radius distribution is presented in Figure S4. It indirectly supports the idea of the self-assembly happening during the synthesis.

## 4. Conclusions

Reversible deactivation radical copolymerization of the monomers with greatly different activity is commonly hard for realization irrespective of the specific mechanism of its implementation. This is due to the different conditions required for the activation (revival) of the propagating species with terminal units of “more activated” or “lower activated” monomers. Nonetheless, in the present work, we successfully synthesized the gradient copolymer of acrylic acid (AA) and vinyl acetate (VAc) using asymmetrical trithiocarbonate based on polyethylene oxide. RAFT copolymerization of AA and VAc proceeded in the mixed solvent—1,4-dioxane/water. This solvent is a selective solvent for forming the AA-VAc gradient copolymer since 1,4-dioxane is a good solvent for both poly(AA) and poly(VAc), while water is a good solvent for poly(AA) and poor solvent for poly(VAc). This leads to the self-assembly of the growing copolymer chain directly in the polymerization mixture.

Moreover, the obtained gradient copolymers showed a strong tendency for self-organization in the water in micelles. This ability was found to be controlled by the gradient degree of the copolymer, which in turn depends on the monomer feed and the monomer conversion. Finally, the gradient copolymer containing 80% of AA forms the narrowly dispersed micelles in an aqueous solution with an average hydrodynamic radius of about 250 nm. Such micelles with soft poly(VAc) hydrophobic core and poly(AA) hydrophilic corona can be further utilized as containers for the delivery of hydrophobic substances or as nanoreactors. In addition, the known ability of PAA to react upon pH change may lead to the pH sensitivity of the formed micelles.

## Figures and Tables

**Table 1:** Formulations of reaction mixtures for copolymerization.

VAc/AA, mole/mole	VAc		AA		1,4-Dioxane	Water	PSK		PEG-TC	
	mL	mole	mL	Mole	mL	mL	mg	mole	mg	Mole
43/57	6.0	0.065	6.0	0.087	10	14	6.6	2.4 × 10^−5^	129	2.4 × 10^−5^
55/45	7.4	0.080	4.6	0.067	10	14	6.6	2.4 × 10^−5^	129	2.4 × 10^−5^
70/30	9.1	0.099	2.9	0.042	10	14	6.6	2.4 × 10^−5^	129	2.4 × 10^−5^

**Table 2:** Composition of the copolymer of AA and VAc synthesized in PEG-TC mediated copolymerization from various monomer feed.

f_AA_, mole%	Conversion,%	<F_AA_> *, mole%
57	28.0 59.0 77.0	89 82 80
45	57.0	75
30	33.7	65

* <F_AA_>—the average molar part of AA in the copolymer determined by ^1^H NMR spectroscopy.

## Supplementary Materials

The following supporting information can be downloaded at: https://www.mdpi.com/article/10.3390/polym14030555/s1, Figure S1: 1H NMR spectra in DMSO-d6 of the copolymers of VAc and AA synthesized in PEG-TC mediated copolymerization from various monomer feeds; Figure S2: TEM microphotographs of the thin film of the copolymer synthesized through RAFT copolymerization of vinyl acetate and acrylic acid at fAA = 45 mol.% at 57% of monomer conversion; Figure S3: Structure factor of the reaction mixture of vinyl acetate and acrylic acid, fAA = 57 mole%, after 300 min of copolymerization at 80oC, obtained by SAXS method; Figure S4: Hydrodynamic radius Rh distribution of gradient copolymer (fAA = 57 mole%) at conversion 77 in 0.1 wt% in dioxane/water solvent mixture; Table S1: Molecular weights of the copolymers grown in copolymerization of acrylic acid and vinyl acetate in the presence of PEG-TC.
