# Supplementary material for: RAFT Copolymerization of Vinyl Acetate and Acrylic Acid in the Selective Solvent
Source: Polymers (Basel). 2022 Jan 29;14(3):555. doi: 10.3390/polym14030555 (PMC8838437; doi:10.3390/polym14030555)
Supplement: Supplementary file 1 [file polymers-14-00555-s001.zip › polymers-1574114-supplementary.pdf]

Supplementary Data for

RAFT copolymerization of vinyl acetate and acrylic acid in the selective solvent

Elena Yu. Kozhunova[1,2*], Anna V. Plutalova[2], Elena V. Chernikova[2*]

[1]Lomonosov Moscow State University, Faculty of Physics, Russian Federation, 119991, Moscow, Lenin Hills, 1, bld. 2

[2]Lomonosov Moscow State University, Faculty of Chemistry, Russian Federation, 119991, Moscow, Lenin Hills, 1, bld. 3

[*] Correspondence: kozhunova@polly.phys.msu.ru (EK), chernikova_elena@mail.ru (EC)

Table of contents

Experimental 2

Figure S1. ^{1}H NMR spectra in DMSO-d6 of the copolymers of VAc and AA synthesized in PEG-TC mediated copolymerization from various monomer feeds. 4

Figure S2. TEM microphotographs of the thin film of the copolymer synthesized through RAFT copolymerization of vinyl acetate and acrylic acid at f_{AA} = 45 mol. % at 57 % of monomer conversion. 6

Figure S3. Structure factor of the reaction mixture of vinyl acetate and acrylic acid, f_{AA} = 57 mole %, after 300 min of copolymerization at 80°C, obtained by SAXS method. 7

Figure S4. Hydrodynamic radius R_h distribution of gradient copolymer (f_{AA} = 57 mole %) at conversion 77 in 0.1 wt% in dioxane/water solvent mixture. 8

Table S1. Molecular weights of the copolymers grown in copolymerization of acrylic acid and vinyl acetate in the presence of PEG-TC. 9

Experimental

TEM

The microphotographs of the dispersions were obtained using a Leo 912 AB Omega (Karl Zeiss) transmission electron microscope (TEM) operating at an accelerating voltage of 100kV. 1 μl of the diluted emulsion solutions were deposited on carbon and formvar–coated copper TEM grids and dried at room temperature.

SAXS

X-ray synchrotron radiation small-angle scattering data were collected at the BioSAXS station (Kurchatov synchrotron radiation source) with the energy of incident beam 8 keV ($\lambda = 1.445$ Å) using a 2D DECTRIS Pilatus3 1M photon-counting detector [1]. The beam was collimated using a system of four slits and focused on the detector by monochromator in the horizontal dimension and by a mirror in the vertical dimension. The size of the beam on the detector was approximately 0.5×0.35 mm. Preliminary instrument calibration was performed just before the experiments using a silver behenate sample with an exposure of 60 seconds. The sample-to-detector distance was 2500 mm that provided a scattering vector range from 0.003 to 1.5 nm−1. Polymerization mixture without purification was placed in a cylindrical capillary 5 cm in length and 2 mm in diameter. Measurement was performed at 25 °C. The data were normalized to the intensity of the transmitted beam and radially averaged. The solvent scattering was subtracted, and the curve was scaled using the PRIMUS program from ATSAS software suite. Hard sphere model was used as a fitting model for evaluation of the inhomogeneities size [2].

References

1.G.S. Peters, O.A. Zakharchenko, P.V. Konarev, Y.V. Karmazikov, M.A. Smirnov, A.V. Zabelin, E.H. Mukhamedzhanov, A.A. Veligzhanin, A.E. Blagov, M.V. Kovalchuk, The small-angle X-ray scattering beamline BioMUR at the Kurchatov synchrotron radiation source, Nucl. Instruments Methods Phys. Res. Sect. A Accel. Spectrometers, Detect. Assoc. Equip. 945 (2019). doi:10.1016/j.nima.2019.162616.

2. E.Yu. Kozhunova, V.Yu. Rudyak, X. Li, M. Shibayama, G.S. Peters, O.V. Vyshivannaya, I.R. Nasimova, A.V. Chertovich, Microphase separation of stimuli-responsive interpenetrating network microgels investigated by scattering methods, Journal of Colloid and Interface Science, 2021, vol. 597, pp. 297-305,

Figure S1. ^{1}H NMR spectra in DMSO-d6 of the copolymers of VAc and AA synthesized in PEG-TC mediated copolymerization from various monomer feeds.

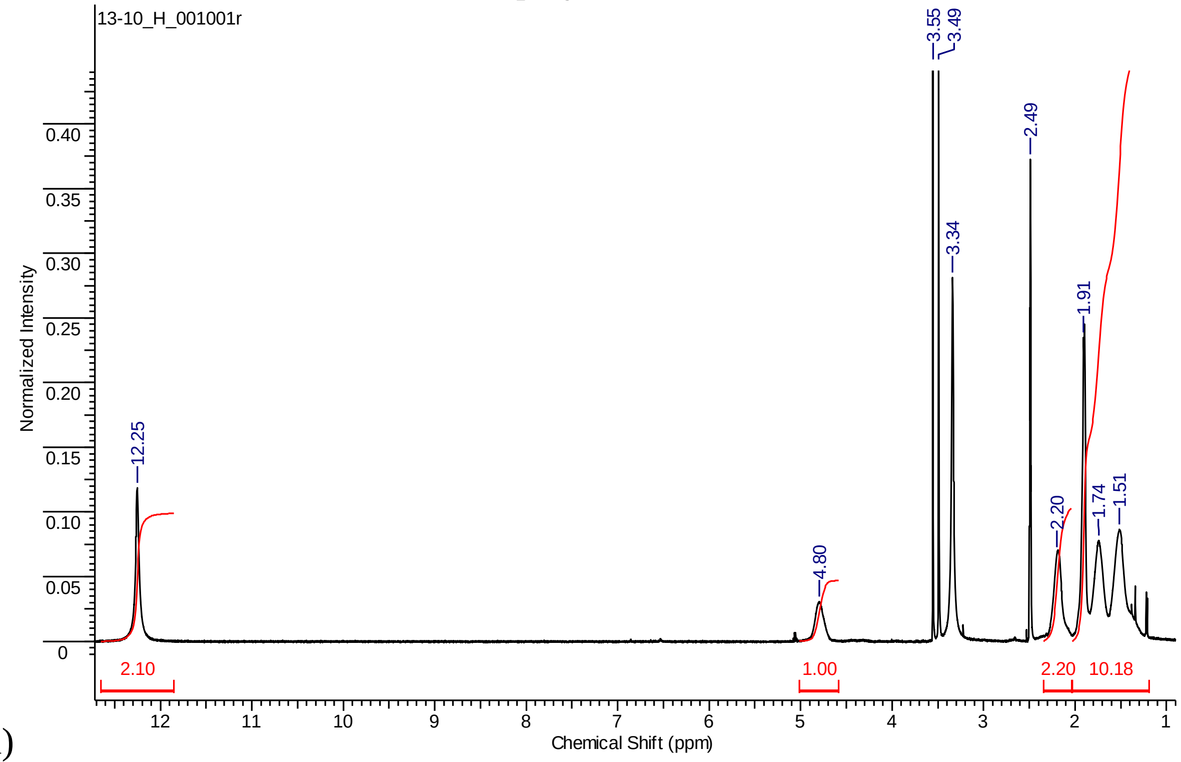

(a)

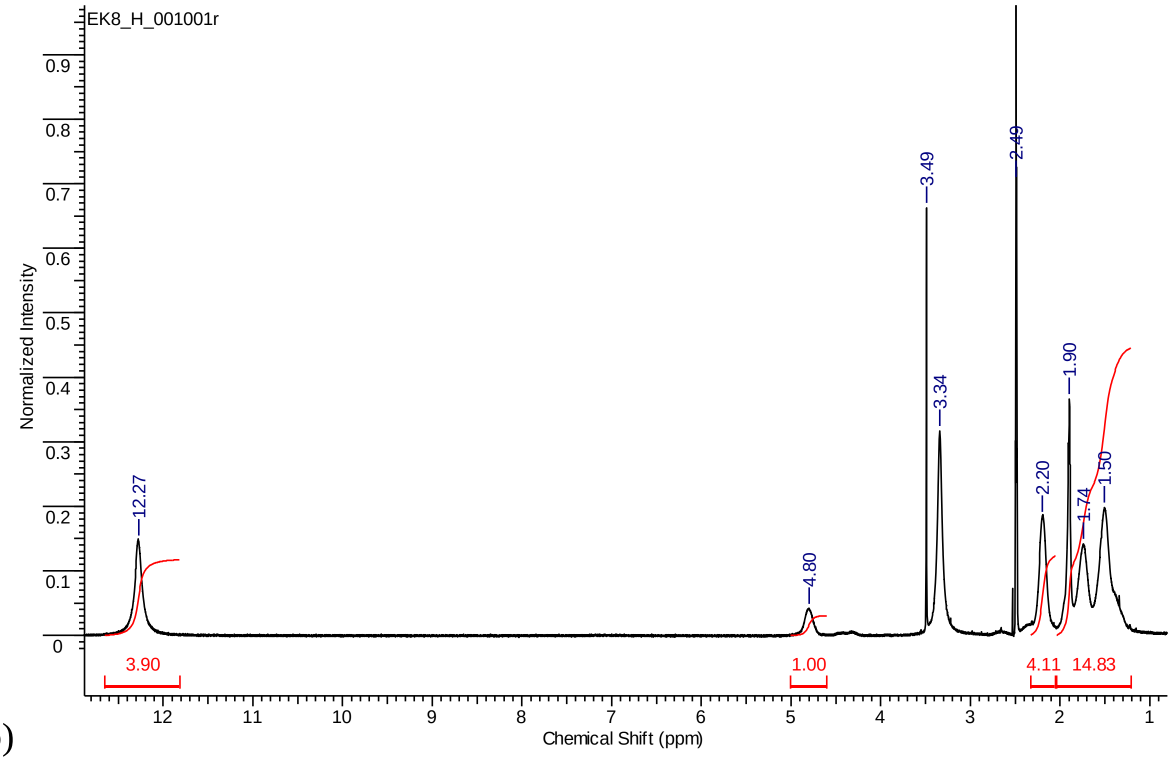

(b)

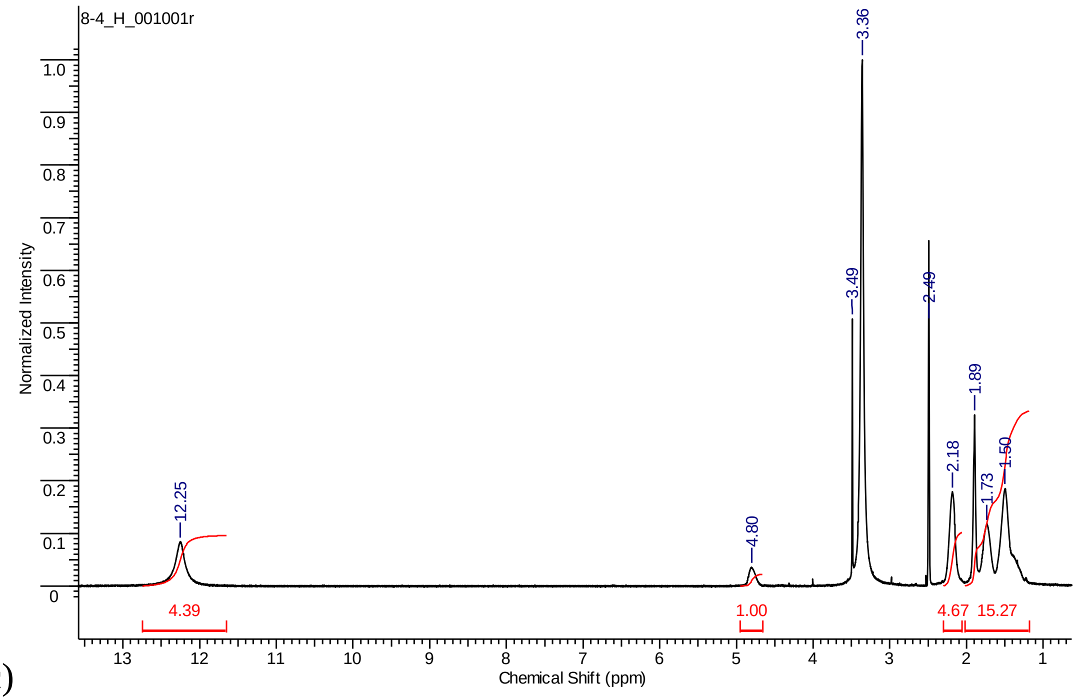

(c)

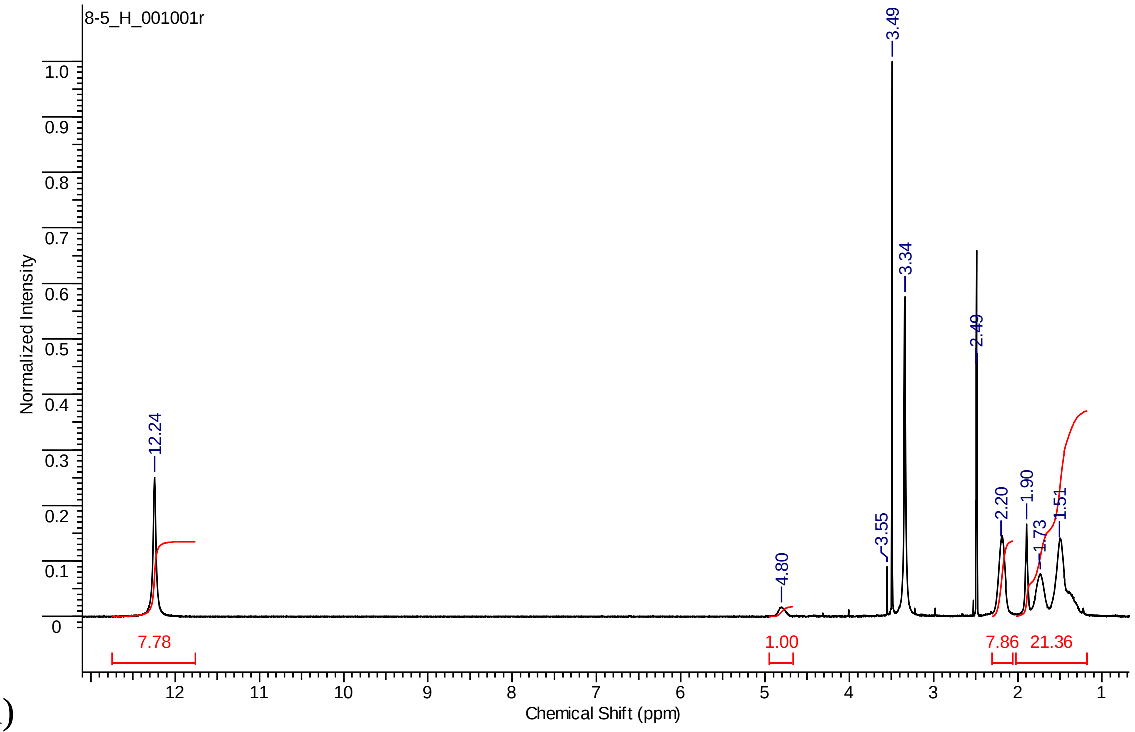

(d)

The peaks at 3.34 and 3.49 ppm correspond to methoxy (CH_3O) and methylenoxy (CH_2O) protons of poly(ethylene glycol) methyl ether substituent. The peak at 2.20 ppm can be assigned to CH protons of AA units of the main chain. The peak at 4.80 ppm can be referred to CH protons of VAc units of the main chain. The signals in the 1.51 – 1.90 ppm region correspond to the methylene (CH_2) protons of the main chain, methyl protons of VAc units, and methyl and methyle protons of $C_{12}H_{25}$ substituent. The signal at 2.49 ppm is assigned to DMSO-d6, and the signal at 3.55 ppm is referred to as the residual water.

Figure S2. TEM microphotographs of the thin film of the copolymer synthesized through RAFT copolymerization of vinyl acetate and acrylic acid at f_{AA} = 45 mol. % at 57 % of monomer conversion.

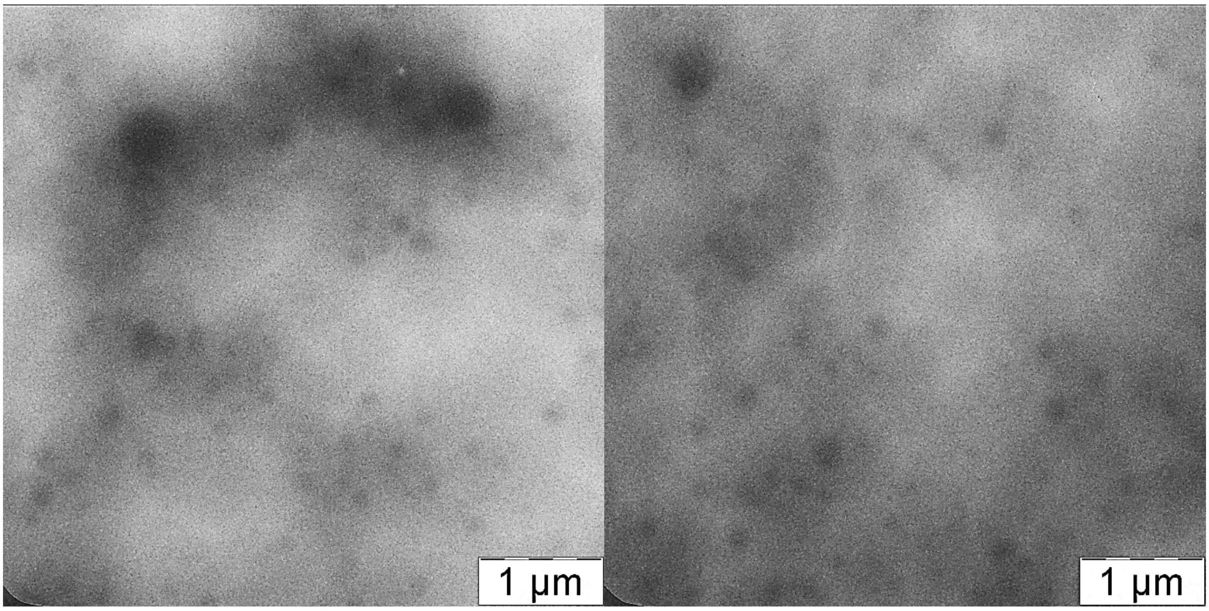

Figure S2 shows inhomogeneities (dark round objects with the size of about 100 nm) seen in the dried film of the copolymer synthesized through RAFT copolymerization of AA and VAc, presumably the hydrophobic cores of the formed micelles. Further investigation is needed, preferably using the cryo-TEM method.

Figure S3. Structure factor of the reaction mixture of vinyl acetate and acrylic acid, f_{AA} = 57 mole %, after 300 min of copolymerization at 80°C, obtained by SAXS method.

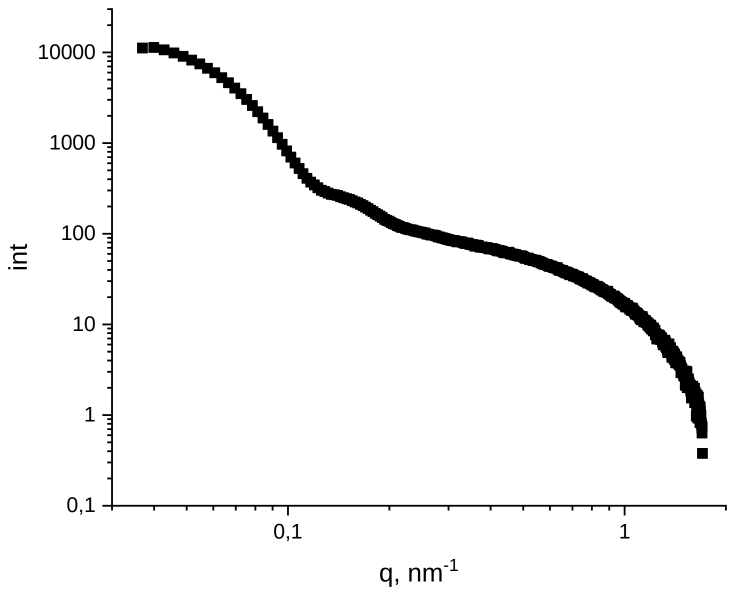

Figure S3 shows that the reaction mixture at the final stages of the copolymerization process contains aggregates of the size of about 40-60 nm, which could be due to the polymerization-indices self-assembly of growing gradient polymer chains of VAc and AA in the selective solvent.

Figure S4. Hydrodynamic radius R_h distribution of gradient copolymer (f_{AA} = 57 mole %) at conversion 77 in 0.1 wt% in dioxane/water solvent mixture.

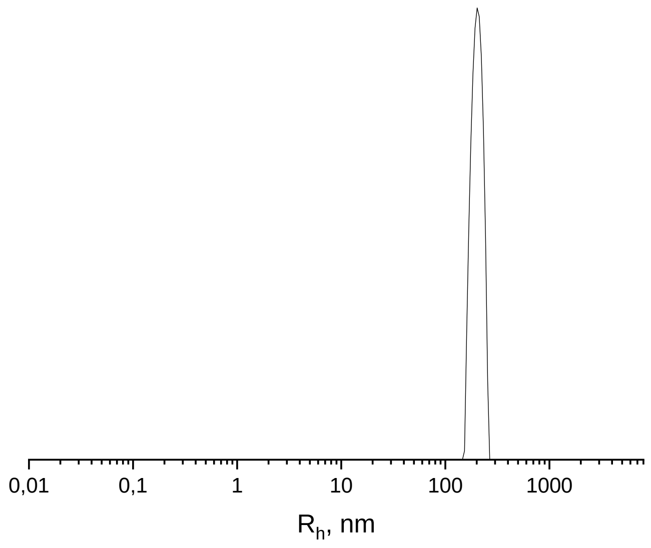

Figure S4 shows that the obtained gradient copolymer can form aggregates in water/dioxane solution similar to that used during the polymerization process.

Table S1. Molecular weights of the copolymers grown in copolymerization of acrylic acid and vinyl acetate in the presence of PEG-TC.

f_{AA}, mole %	Time, h	Conversion, %	M_n, kg/mole	M_w/M_n
30	5	1.1	19.4	1.19
	10	10.8	63.7	1.36
	20	14.9	81.6	1.38
	40	28.2	93.6	1.42
	60	33	103.6	1.56
45	5	1.5	22.4	1.49
	10	10.8	43.4	1.42
	20	30.1	136.2	1.58
	40	38.3	137.2	1.68
	60	48.0	149.9	1.69
	300	57.0	156.8	1.70
57	10	28	124.3	1.67
	20	59	137.2	1.74
	70	75	161.5	1.78
	300	77	166.5	1.79
